# Building a Machine Learning-based Ambulance Dispatch Triage Model for Emergency Medical Services

**DOI:** 10.34133/hds.0008

**Published:** 2023-03-15

**Authors:** Han Wang, Qin Xiang Ng, Shalini Arulanandam, Colin Tan, Marcus E. H. Ong, Mengling Feng

**Affiliations:** ^1^ Saw Swee Hock School of Public Health, National University Health System, National University of Singapore, Singapore.; ^2^ Singapore Civil Defence Force, Singapore.; ^3^ Health Services Research Centre, Singapore Health Services, Singapore.; ^4^ Health Services and Systems Research, Duke-NUS Medical School, National University of Singapore, Singapore.; ^5^ Department of Emergency Medicine, Singapore General Hospital, Singapore.; ^6^ Institute of Data Science, National University of Singapore, Singapore.

## Abstract

**Background:**

In charge of dispatching the ambulances, Emergency Medical Services (EMS) call center specialists often have difficulty deciding the acuity of a case given the information they can gather within a limited time. Although there are protocols to guide their decision-making, observed performance can still lack sensitivity and specificity. Machine learning models have been known to capture complex relationships that are subtle, and well-trained data models can yield accurate predictions in a split of a second.

**Methods:**

In this study, we proposed a proof-of-concept approach to construct a machine learning model to better predict the acuity of emergency cases. We used more than 360,000 structured emergency call center records of cases received by the national emergency call center in Singapore from 2018 to 2020. Features were created using call records, and multiple machine learning models were trained.

**Results:**

A Random Forest model achieved the best performance, reducing the over-triage rate by an absolute margin of 15% compared to the call center specialists while maintaining a similar level of under-triage rate.

**Conclusions:**

The model has the potential to be deployed as a decision support tool for dispatchers alongside current protocols to optimize ambulance dispatch triage and the utilization of emergency ambulance resources.

## Introduction

Efficient and effective Emergency Medical Services (EMS) are vital for good outcomes during pre-hospital emergencies such as stroke and cardiac arrest, where the response time and the type of response matters [1–3]. The traditional approach that dispatches the closest available vehicle to an emergency has been shown to be far from optimal [4,5]. With limited ambulance resources, the priority of a case is a crucial factor for dispatch decisions [6–9].

EMS systems worldwide have investigated and implemented various pre-hospital triage systems to determine the priority level of a pre-hospital emergency case. Most of these systems can be categorized into 2 groups. The Medical Priority Dispatch System and its variants assign priority levels to each case based on protocols with scripted questions put to the caller [10,11] and originated in North America. On the other hand, Criteria-Based Dispatch systems involve guidelines to determine response levels based on patient signs and symptoms collected by the dispatcher [12,13] and are used, for example, in Nordic and European countries. However, studies have shown that the accuracy of these priority dispatch systems remains an issue of concern [14,15], and there is a paucity of research to guide pre-hospital triage systems [16].

In Singapore, the Singapore Civil Defence Force (SCDF) serves as the national EMS organization, and their ambulance crews respond to more than 190,000 calls (national emergency “995” hotline) every year. With only a fleet of 84 ambulances, the need for efficient resource utilization is pressing, especially as the population continues to grow and age. At present, the SCDF uses a rule-based system containing 30 in-house protocols similar to the Criteria-Based Dispatch system. For different chief complaints, the dispatcher at the call center will ask questions based on the respective protocol and assign a Patient Acuity Category Scale (PACS) [17] for medical dispatch. The PACS is the emergency scale used nationwide in Singapore’s EMS system that includes 5 levels (P1+, P1, P2, P3, and P4). P1+ and P1 are assigned to the most severe cases that are immediately life-threatening, such as cardiac arrest and head injury. P1+ cases will trigger a fire bike that beats the traffic on the road and will arrive faster than an ambulance. P2 cases are emergencies where the patients are usually unable to walk and are in some form of distress. If not attended early, then their medical status could deteriorate quickly. P3 cases are minor emergencies involving patients who have mild to moderate symptoms and are able to walk. Early intervention will still result in a better patient outcome in P3 cases. P4 cases are nonemergencies such as old injuries or chronic conditions that do not require immediate attention.

During an emergency call, the call center specialists will decide the PACS according to the patient’s presenting complaints, symptoms, and the nature of the emergency, allowing a quick identification of the case acuity with no objective measurements available. After the ambulance is dispatched and arrives at the scene, paramedics will decide the PACS guided by a list of presenting complaints and provisional diagnoses, which they arrive at after their more detailed history taking, scene assessment, and physical examination of the patient. A quick overview of the PACS levels is shown in Table 1.

**Table 1.** Details of 5 levels of PACS used in SCDF.

**PACS**	**Definition**	**Response**	**Example**
P1+	Life-threatening emergencies	Highest priority, fastest response (extra resources deployed)	Cardiac arrest
P1	Life-threatening emergencies	Highest priority, fastest response	Head injury
P2	Emergencies	High priority, fast response	Abdominal pain
P3	Minor emergencies	Lower priority, slower response	Persistent diarrhea
P4	Nonemergencies	No response	Cough

The purpose of the call center triage is to determine the (a) need for urgent intervention, (b) need for hospital conveyance, and (c) urgency of conveyance. This is fairly similar to the purpose of field triage by the paramedics, and hence, we seek concurrence between the 2 as much as possible. However, call center specialists, who do not have professional registration but only receive vocational training in SCDF, have a challenging job deciphering caller information and dispatching ambulance support (if necessary) within their required time limit: 1 min. Furthermore, the protocols are designed to allow quick comprehension and utilization but have several limitations. For example, the protocols may not be very specific and do not take other patient-reported information into consideration. Currently, we observed around 47% over-triage rate and 6% under-triage rate by the call center specialists in the period of January 2018 and August 2020 over more than 300,000 cases, using the acuity reported by the paramedics at the scene as ground truth. This means that, for every 100 emergency cases, call center specialists will assign a more-severe PACS (over-triage) in 47 cases and a less-severe PACS (under-triage) in 6 cases than that from the paramedics. We postulated that a machine learning-based model, digesting information captured solely from the call, can improve the overall dispatching effectiveness by reducing over-triage and keeping under-triage at the same level.

There have been some studies exploring machine learning on medical emergency calls. In Copenhagen, Blomberg et al. [18] used a machine learning framework to recognize cardiac arrest in emergency calls, but the details of the machine learning framework is proprietary and, hence, was not disclosed. In the their randomized clinical trial, no significant improvement in dispatchers’ ability to recognize cardiac arrest was found when supported by machine learning [19]. In London, Tollinton et al. [20] used machine learning models to predict whether an unconscious and fainting patient would be conveyed to a hospital using the Medical Priority Dispatch System codes and free text notes as features. However, using conveyance as a binary marker of case severity is neither accurate nor objective.

In this proof-of-concept study, we aimed to develop a machine learning-based model for EMS ambulance dispatch triage in Singapore. The model should be easily applied to a wide range of pre-hospital emergency cases and decrease the level of over-triage without increasing the level of under-triage.

## Methods

### Data collection and preprocessing

All SCDF ambulance dispatch cases between January 2018 and August 2020 that were assigned a PACS of either P1+, P1, P2, P3, or P4 were included. A small number of cases with no meaningful call conversation, as well as cases that fell under a protocol of less than 0.1% prevalence, was excluded. Data were linked using the unique incident number and then subsequently anonymized. All data were retrieved from both the call center record system and ambulance case record system electronically.

We used the PACS reported by paramedics at the scene as the ground truth. In particular, we grouped P1+ and P1 as “critical emergency,” P2 as “normal emergency,” and P3 and P4 as “nonemergency” to represent the case acuity. Critical emergency cases would require the ambulance to be dispatched as soon as possible. Normal emergency cases also required assistance from an ambulance, but the arrival time can be less stringent. Meanwhile, nonemergency cases could be deprioritized when ambulance resources are strained, and future research can explore diverting these cases into other alternative care pathways such as primary care, telemedicine, or outpatient visits.

### Feature engineering

The medical information obtainable from an emergency call is very limited. We chose to include basic patient demographics (age and gender) and the chief medical complaint of the case as predictor variables in our model because these variables were currently being collected by the call center specialists based on the answers and understanding of the situation before the triage decision was made. For cases where the age of the patient was not available, we imputed with the median age of the cohort.

**Fig. 1.**
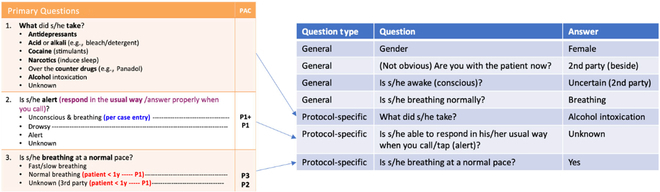
Illustration of a pseudo-dialog derived from an existing protocol.

Because of the urgency of emergency calls, SCDF call center specialists do not note down every single word of the conversation but choose the most appropriate answers for the protocol questions, with the rule-based system behind prompting the PACS. Such pseudo-dialogs consisting of question–answer (QA) pairs were the only available representation of the actual conversation, containing important information like the patient’s consciousness and breathing. An example of a protocol and a pseudo-dialog are shown in Fig. 1. On the left-hand side, it shows the protocol that call center specialists need to follow when the nature of the emergency is “poisoning/ingestion.” Specific questions such as what the patient took, whether the patient is alert, and whether the patient was breathing normally were asked to decide the PACS. On the right-hand side, it shows the pseudo-dialog consisting of 7 QA pairs extracted from an alcohol intoxication case, where the first 4 questions were from a complaint-agnostic general protocol and the last 3 questions were from the “poisoning/ingestion” protocol. According to the answers, this case would be considered as P3.

**Fig. 2.**
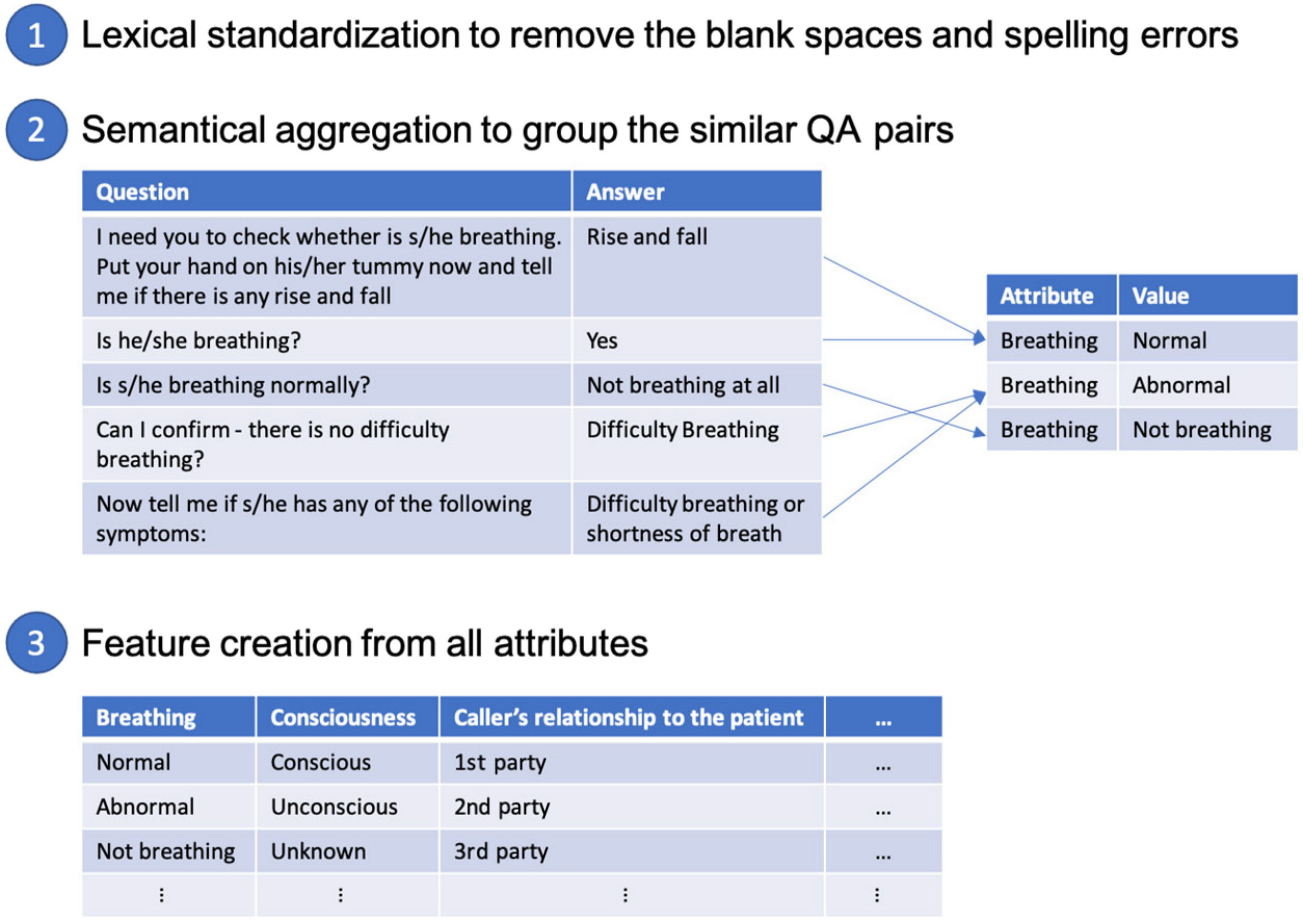
Illustration of the process to transform the QA pairs into the final features.

To tackle this challenge, we proposed the following feature engineering approach. First, all unique QA pairs in our dataset were extracted, and each group of lexically similar questions was standardized to one question by removing the blank spaces and spelling errors. The lexical similarity of the questions was measured by Levenshtein distance [21]. Second, all QA pairs were manually screened and grouped into mutually exclusive attributes and values that are semantically meaningful. Last, features were created from these attributes with ordinal encoding and used in the predictive model. The step-by-step process of creating these features is shown in Fig. 2. For each emergency call, if the attribute was asked more than once, then the latest value was used. If the attribute was never asked, then a “not-asked” value was assigned to the attribute.

### Bayesian optimization for class weights

**Fig. 3:**
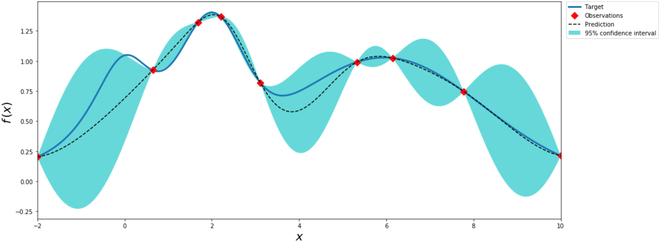
An illustration of Bayesian optimization.

An under-triage would cause significantly more harm than an over-triage, which is why the existing rule-based system was designed to tend to over-triage patients. However, machine learning models, by default, would not know the implied difference between an under-triage and an over-triage, resulting in undesirable similar rates of the 2 errors. To control the under-triage rate to be similar to that of the call center specialists, we tuned the class weight of critical emergency cases using Bayesian optimization [22]. As illustrated in Fig. 3, Bayesian optimization approximates the objective function (represented by the blue curve) based on a posterior distribution of Gaussian processes (represented by the dashed line and the cyan areas) with observations (represented by the red points).

We designed the following objective function *F*, where *f*(*x*) represents the overall under-triage rate yielded by the machine learning model given a class weight of under-triage rate represented by *x*, and 0.0684 is the under-triage rate of the call center specialists.

$$F=\max_{x}-{\left(f(x)-0.0684\right)}^{2}$$

After the fitting process, we could obtain an *x* that maximizes *F* so that the overall under-triage rate from the model is close to the under-triage rate of the call center specialists. We ran 30 iterations with 5 initial points and constrained *x* to be between 1 and 10 for each model. The class weights for normal emergency and nonemergency were kept as 1 for simplicity.

### Models and experimental setup

We randomly generated 10 different splits, each having 80% as training data and 20% as testing data, from our dataset. In each split, we experimented with different machine learning models, namely, Logistic Regression, Decision Tree [23], Extreme Gradient Boosting (XGBoost) [24], and Random Forest [25], together with the class weights derived by our Bayesian optimizations to control the under-triage rates. Except for age, all attributes were treated as categorical features in the model. We compared the model predictions to the PACS assigned by the call center specialists. We reported the 4 metrics that we would like to improve on: overall over-triage rate, triage accuracy, and likelihood ratios of critical emergency cases and nonemergency cases. The mean and 95% confidence interval of the metrics were estimated with the metrics from the 10 splits. All data processing and statistical analysis were carried out in Python 3.8 using libraries including pandas, scikit-learn, and bayes_opt.

## Results

**Fig. 4.**
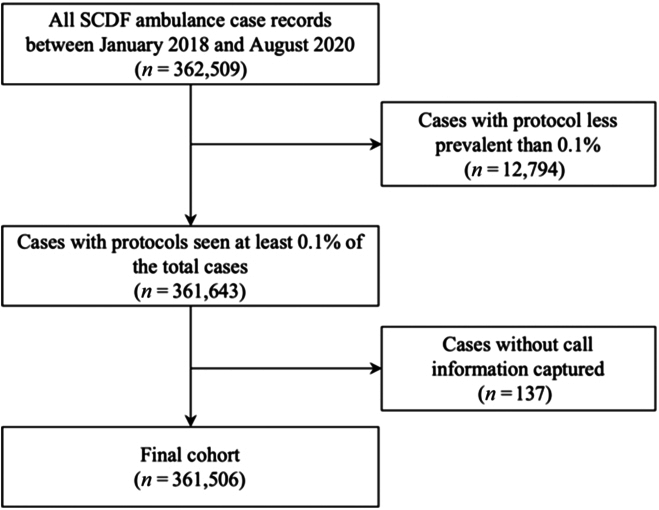
Cohort selection flow chart.

The process to select the final cohort of 361,506 cases is shown in Fig. 4, and the demographics and most frequently asked attributes of the cohort are shown in Table 2. Among the final cohort, 191,474 (58.4%) of the patients were male, and the median age was 61 years old (interquartile range, IQR 41–77). In some cases, the age (13.0%) and the gender (9.3%) of the patient were not collected because of either nonresponse from a second or third party caller or data entry error.

**Table 2.** Characteristics of the cohort grouped by the case severity.

**Characteristics**	**Total**	**Critical**	**Emergency**	**Nonemergency**
*n* (%)	361,506	54,134	233,886	73,486
Age				
Not asked, *n* (%)	46,853 (13.0)	7,127 (13.2)	25,986 (11.1)	13,740 (18.7)
Asked, median [Q1,Q3]	61.0 [41.0,77.0]	69.0 [54.0,80.0]	64.0 [45.0,78.0]	49.0 [30.0,66.0]
Gender, *n* (%)				
Female	136,512 (37.8)	19,448 (35.9)	93,639 (40.0)	23,425 (31.9)
Male	191,474 (53.0)	29,981 (55.4)	120,868 (51.7)	40,625 (55.3)
Not asked	33,520 (9.3)	4,705 (8.7)	19,379 (8.3)	9,436 (12.8)
Caller identity, *n* (%)				
First party	27,870 (8.1)	1,543 (3.0)	17,211 (7.7)	9,116 (13.0)
Second party	272,987 (79.1)	43,790 (84.8)	181,043 (81.0)	48,154 (68.7)
Third party	44,410 (12.9)	6,325 (12.2)	25,255 (11.3)	12,830 (18.3)
Consciousness, *n* (%)				
Not conscious	61,922 (17.1)	17,927 (33.1)	36,375 (15.6)	7,620 (10.4)
Conscious	244,972 (67.8)	27,219 (50.3)	166,343 (71.1)	51,410 (70.0)
Unknown	6,604 (1.8)	1,190 (2.2)	4,057 (1.7)	1,357 (1.8)
Not asked	46,462 (12.9)	7,688 (14.2)	26,015 (11.1)	12,759 (17.4)
Not applicable	1,546 (0.4)	110 (0.2)	1,096 (0.5)	340 (0.5)
Breathing, *n* (%)				
Not breathing	4,681 (1.3)	3,187 (5.9)	1,202 (0.5)	292 (0.4)
Abnormal breathing	29,703 (8.2)	7,600 (14.0)	18,913 (8.1)	3,190 (4.3)
Normal breathing	229,504 (63.5)	27,564 (50.9)	155,056 (66.3)	46,884 (63.8)
Unknown	4,265 (1.2)	783 (1.4)	2,673 (1.1)	809 (1.1)
Not asked	93,353 (25.8)	15,000 (27.7)	56,042 (24.0)	22,311 (30.4)
Bleeding, *n* (%)				
Not bleeding	88,098 (24.4)	6,320 (11.7)	60,611 (25.9)	21,167 (28.8)
Bleeding	18,333 (5.1)	1,017 (1.9)	11,925 (5.1)	5,391 (7.3)
Unknown	6,169 (1.7)	756 (1.4)	4,045 (1.7)	1,368 (1.9)
Not asked	248,906 (68.9)	46,041 (85.1)	157,305 (67.3)	45,560 (62.0)
Ability to speak, *n* (%)				
No	27,313 (7.6)	6,915 (12.8)	17,620 (7.5)	2,778 (3.8)
Yes	49,335 (13.6)	7,187 (13.3)	34,525 (14.8)	7,623 (10.4)
Unknown	1,656 (0.5)	390 (0.7)	1,072 (0.5)	194 (0.3)
Not asked	276,365 (76.4)	37,372 (69.0)	176,580 (75.5)	62,413 (84.9)
Not applicable	6,837 (1.9)	2,270 (4.2)	4,089 (1.7)	478 (0.7)

The performance of our models versus the call center specialists on the training data is shown in Table 3. The Random Forest model was chosen as the final model because of its performance, achieving an accuracy of 63.7% and an over-triage rate of 29.6%, significantly outperforming current call center protocols by an absolute margin of around 15%. Both likelihood ratios were also significantly higher than the call center specialists.

**Table 3.** Performance metrics of various models versus the baseline of call center protocols on the training data.

	**Accuracy**	**Over-triage**	**Under-triage**	**Critical likelihood ratio**	**Nonemergency likelihood ratio**
Logistic Regression	52.4%[52.3% to 52.4%]	40.8%[40.7% to 40.8%]	6.9%[6.9% to 6.9%]	2.0[2.0–2.0]	3.7[3.7–3.7]
Decision Tree	57.1%[56.0% to 58.1%]	36.8%[35.6% to 38.0%]	6.1%[5.9% to 6.4%]	2.5[2.4–2.6]	8.5[7.9–9.1]
XGBoost	59.6%[59.5% to 59.6%]	33.6%[33.5% to 33.7%]	6.8%[6.8% to 6.9%]	3.0[2.9–3.0]	6.3[6.3–6.4]
Random Forest	63.7%[63.6% to 63.9%]	29.6%[29.4% to 29.7%]	6.7%[6.6% to 6.8%]	3.7[3.6–3.7]	14.7[14.2–15.2]
Call center specialists	47.7%	45.5%	6.8%	1.8	2.6

Comparing the performance on test data shown in Table 4 with the performance on training data, we could also observe that the models performed consistently and did not suffer from overfitting.

**Table 4.** Performance metrics of various models versus the baseline of call center protocols on the test data.

	**Accuracy**	**Over-triage**	**Under-triage**	**Critical likelihood ratio**	**Nonemergency likelihood ratio**
Logistic Regression	52.3%[52.1% to 52.4%]	40.8%[40.7% to 41.0%]	6.9%[6.9% to 7.0%]	2.0[2.0–2.0]	3.7[3.7–3.8]
Decision Tree	54.7%[53.8% to 55.6%]	37.9%[36.7% to 39.1%]	7.4%[7.1% to 7.7%]	2.3[2.2–2.4]	3.9[3.7–4.1]
XGBoost	58.6%[58.5% to 58.8%]	34.0%[33.9% to 34.2%]	7.3%[7.2% to 7.4%]	2.8[2.8–2.8]	5.5[5.3–5.6]
Random Forest	60.8%[60.7% to 60.9%]	31.1%[31.0% to 31.2%]	8.1%[8.0% to 8.1%]	3.2[3.2–3.2]	5.3[5.2–5.3]
Call center specialists	47.6%	45.7%	6.7%	1.8	2.7

**Fig. 5.**
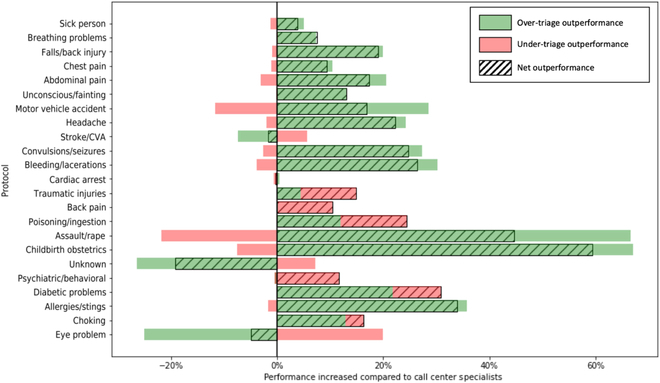
Difference in performance between our model and the call center specialists on test data stratified by the different protocols.

The difference in performance between our model and the call center specialists on test data stratified by the different protocols is shown in Fig. 5. Compared to the call center specialists, the over-triage rates achieved by our model were better in most of the protocols (represented by green bars to the right of the 0% vertical line), while the under-triage rates increased/decreased, varying across different protocols (represented by red bars to the left/right of the 0% vertical line). Overall, the accuracy in most of the protocols was improved (represented by bars filled with slanted lines to the right of the 0% vertical line).

**Fig. 6.**
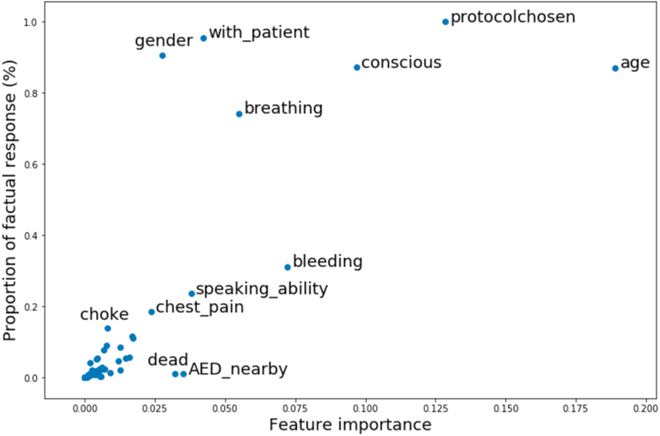
Illustration of the proportion of factual response versus the feature importance derived for each feature.

The proportion of factual response versus the feature importance is shown in Fig. 6. A factual response meant that the value of the feature was extracted from the QA pairs instead of the “not-asked” value. The closer the proportion of real response is to 100, the more frequent the information was asked. The age had the highest importance among all the features. Other important features include the protocol chosen; whether the patient was conscious, bleeding, breathing, and able to speak were also well expected. On the contrary, we found that the information of whether the patient was experiencing chest pain did not contribute as much to the model, although chest pain was one of the priority symptoms together with breathing, consciousness, and bleeding in the protocol that would be automatically assigned as P1+.

## Discussions

In this study, we used emergency call center and ambulance case records to develop a machine learning-based multiclass classification model to improve ambulance dispatch triage performance for Singapore EMS. With no extra data/information compared to what is currently available to dispatchers, our model outperformed current baseline performance by an absolute margin of ~15% in terms of reducing over-triage while maintaining a similar level of under-triage. This model has the potential to be deployed as a decision support tool alongside existing protocols to optimize pre-hospital triage and utilization of emergency ambulance resources. Because EMS systems differ from country to country, our model may not be directly applicable to others. However, the importance of our work lies in demonstrating a methodology for processing EMS call center data and developing a machine learning model, which could be generalized to other EMS systems worldwide. The sharing of our actual protocol and data could be requested and will be subjected to the approval from the EMS system in Singapore.

### Ambulance dispatch triage with machine learning

Recently, machine learning models have been applied to several fields in EMS including symptom recognition [18], survival prediction [3], patient conveyance [20], paramedic documentation audit [26,27], emergency department (ED) record linkage [28], etc. However, no previous study has applied machine learning in optimizing pre-hospital triage, despite the fact that the accuracy of the medical dispatching systems are concerning [15]. To our knowledge, our study is the first to address ambulance dispatch triage optimization with machine learning, and we hope that our study inspires more research in this direction. With multimodal data available for EMS in the future (e.g., real-time video, previous patient health record, and remotely monitored vital signs), machine learning and deep learning could certainly transform EMS to a whole new level.

### Hypothesis of lower feature importance of chest pain

Currently in SCDF, callers that report any priority symptoms (abnormal breathing, chest pain, decreased consciousness, or profuse (nonstop) bleeding) would be automatically assigned P1+. Our model showed that breathing, consciousness, and bleeding were indeed important indicators of the case acuity, while chest pain was less reliable. Chest pain is an exceedingly common complaint in emergency departments worldwide, and oftentimes, the most common causes are relatively benign, e.g., musculoskeletal conditions, gastrointestinal disease, or stable coronary artery disease [29]. Therefore, chest pain alone might not be specific enough in determining the acuity of the case.

### Limitations

Our study has several limitations. First, this was a single-center study using retrospective data. A future prospective study implementing a decision support system using this model is required to validate our approach internally, and studies in other centers with similar EMS systems are required to validate our methodology externally. Second, we used PACS reported by the paramedics to derive the ground truth instead of the final PACS assessed in the ED because of unavailability of hospital data. Although the paramedics have more information to work with and are trained in making a sound medical assessment, the eventual PACS could still change between the time of ambulance dispatch and the time of ambulance arrival at the emergency department. However, it might be debatable whether paramedics or the ED triage nurses have a better assessment of the case acuity at that specific point in time. We also found that, in Singapore and globally, there is a lack of evidence on the consistency of triage judgment made by the field paramedics and ED triage nurses. It will be desirable for future studies to investigate this issue when the data become available. Third, the information we used in the model was limited to the current call. We have plans to include the patient’s historical emergency call information and health record to provide an even better triage prediction in our future studies. Fourth, similar to retrospective analysis on hospital electronic health records, our study was a secondary analysis of call center records. Hence, the data were subject to human error, especially given the urgent nature of the call center specialists’ job. As with most large cohort studies, there were also varying amounts of missing data that was excluded from analysis. Fifth, the model achieved a lower over-triage rate across most of the protocols as shown in Figure 5. Although we maintain the overall under-triage rate the same as the call center specialists, what could be concerning was that the model did not maintain the same level of under-triage rate across some of the most common protocols, such as sick person, falls/back injury, chest pain, and abdominal pain. There is always a trade-off between over-triage and under-triage, and the current protocol has an intentional bias toward not under-triaging patients. While an under-triage may directly put the patient’s life in danger and should be avoided as much as possible, an over-triage may unnecessarily take up health care resources and delay the dispatch for critical emergency cases, indirectly costing lives. Thus, further cost–benefit analysis should be conducted to study a reasonable trade-off, and protocol-specific models could be explored in the future as well. Sixth, as we only had pre-hospital data, we lacked information on the precise etiology of the various conditions and the patients’ overall outcomes, which would have further enriched our analysis. Last, for EMS systems that do not have machine learning capabilities, our approach will be more difficult to adopt. We suggest the EMS systems to find collaborators in local universities or work with us to develop one for their own.

### Conclusion

In conclusion, in this proof-of-concept study, we developed a machine learning model that can reduce over-triage rates significantly while maintaining a similar level of under-triage. These results are encouraging and show that this approach could be used in the call center to provide better ambulance dispatch triage and case acuity recommendation to optimize ambulance resource utilization. The methodology reported in this paper could also be generalized to other EMS centers to develop their own model.

## Data Availability

Data are available upon request and subjected to the approval of the authors’ institutions.

## Supplementary Materials

Supplementary Materials S1. TRIPOD checklist
